# A simplified system for the detection of antennal responses to host-borne volatile organic compounds in sand flies

**DOI:** 10.1186/s13071-025-06998-3

**Published:** 2025-08-19

**Authors:** Onofrio Marco Pistillo, Priscilla Farina, Marcos Antonio Bezerra-Santos, Ilaria D’Isita, Petr Volf, Domenico Otranto, Giovanni Benelli, Giacinto Salvatore Germinara

**Affiliations:** 1 Department of Agricultural Sciences, Food, Natural Resources and Engineering, University of Foggia, Foggia, Italy; 2 Department of Agriculture, Food and Environment, University of Pisa, Pisa, Italy; 3 Department of Veterinary Medicine, University of Bari, Valenzano, Italy; 4 Department of Parasitology, Faculty of Science, Charles University, Prague, Czech Republic; 5 Department of Veterinary Clinical Sciences, City University of Hong Kong, Hong Kong, China

**Keywords:** Electroantennography, HS-SPME, Psychodidae, Phlebotominae, *Leishmania infantum*, Leishmaniasis, Semiochemicals

## Abstract

**Background:**

*Phlebotomus* (*Larroussius*) *perniciosus* (Diptera: Psychodidae) is the most common and predominant vector of *Leishmania infantum* in the Western Mediterranean region. Volatile organic compounds (VOCs) produced by vertebrates are important cues affecting the behaviour of blood-feeding insects. Generally, the identification of putative behaviourally active VOCs involves three distinct phases: extraction, chemical characterization and chemoreceptivity evaluation using electrophysiological techniques. Here, we present a simplified gas chromatography–mass spectrometry–electroantennographic detection (GC–MS–EAD) setup adapted for screening bioactive compounds in sand flies, in which the chemical identification and antennal responses are recorded simultaneously.

**Methods:**

The method integrates: (i) a flow-splitter that balances the flow rate of the two outgoing streams, (ii) GC columns with different lengths and diameters in the two sections splitter-MS and splitter-EAD and (iii) an antennal signal amplifier. The GC–MS–EAD analysis was applied to headspace solid-phase microextraction (HS-SPME) extracts from a healthy dog, and antennal responses were recorded in female *P. perniciosus* sand flies.

**Results:**

The canine VOC profile was predominantly composed of aldehydes, with hexanal and nonanal eliciting the strongest antennal responses in *P. perniciosus*.

**Conclusions:**

This simplified GC–MS-EAD system shows promise for broader application in the study of host–vector interactions. Its use across different host–vector pairs may enhance our understanding of these relationships and inform the development of strategies for integrated vector monitoring and control.

**Graphical Abstract:**

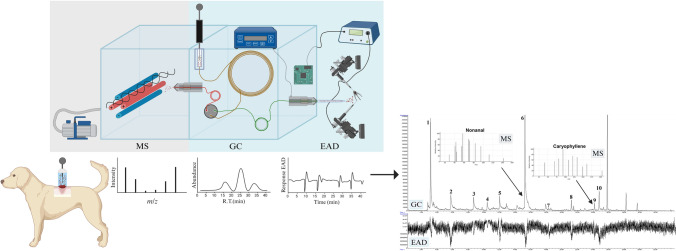

## Background

Phlebotomine sand flies (Diptera: Psychodidae) are vectors of pathogens (i.e., viruses, bacteria and protozoa) and are mostly known for being vectors of *Leishmania spp.* (Kinetoplastida: Trypanosomatidae) to many animal species, including humans [1, 2]. These parasites are the causative agents of leishmaniases, which may occur with three clinical presentations, namely visceral (VL), cutaneous and mucocutaneous [1, 3]. The visceral form, characterised by fever, weight loss, skin lesions and enlargement of the liver, spleen and lymph nodes, can be fatal when not adequately treated [4]. In the Mediterranean basin, the Middle East, Latin America and China, VL is caused by *Leishmania infantum*, which has as primary reservoirs domestic dogs [5] and, occasionally, cats [6], wild leporids [7] and rats [8, 9], among other wildlife species [10, 11]. Moreover, human and animal migrations, urbanisation, ecotourism and climate change are important drivers behind the global spread of VL [12, 13].

*Phlebotomus* (*Larroussius*)* perniciosus* (Newstead) is the most common and predominant vector of *L. infantum* in the Western Mediterranean region [3], where female sand flies take their blood meal chiefly from dogs but also humans, cats, equids and wild carnivores, thus greatly influencing the epidemiology of leishmaniasis in those areas [14, 15]. Sand flies, similarly to any other parasitic arthropod vector (e.g., mosquitoes, triatomines, ticks), locate their hosts using visual, acoustic, thermal and, at close range, olfactory cues (as reviewed by Bezerra‑Santos et al. [16]). Specifically, all vertebrates produce volatile organic compounds (VOCs), in this case also qualifiable as semiochemicals, which are perceived by blood-feeding arthropods and somehow affect their host-seeking behaviour [16]. Notably, some scientific evidence suggests that animals affected by leishmaniasis release VOCs more attractive to sand flies under both laboratory and field conditions [17, 18]. A VOC emission can be collected using different techniques, including headspace solid-phase microextraction (HS-SPME) of an entire individual or part of it. Recognizing putative behaviourally active VOCs requires the identification of compounds in an extract that can stimulate the sensory cells located in the antennal sensilla of the target species. To this aim, the so-called GC-EAD technique (gas chromatography-electroantennographic detection), which combines a gas chromatographic separation with antennal detection, is frequently used. When performing GC-EAD analyses of extracts with unknown composition, chemical characterization by gas chromatography (GC) coupled with mass spectrometry (MS) analysis [19, 20] is necessary. However, comparison of chromatograms recorded with the two different setups can be challenging and time-consuming, thus raising the demand for a coupled GC–MS–EAD technique [21]. The main advantage of this method is the simultaneous recording of compound identity and antennal perception.

The actual effect on the vector could then be corroborated by biological assays (i.e., in Y-tube or wind tunnel olfactometric trials) to determine if such EAD-active compounds are attractive, repellent or neutral [22].

Ideally, the combined and simultaneous use of chemical and electroantennographic detectors (EAD) to record the GC profile and mass spectrum of each peak, along with the insect antennal response through a GC–MS–EAD system, would be of great advantage for research. GC–MS–EAD setups have been used in previous studies. However, this method may have technical limitations related to the transfer speed of the different VOCs when moving towards the mass spectrometer on one side (under vacuum) and the antennal preparation on the other (under atmospheric pressure) [23]. Another hindrance may be represented by the lack of devices built for this purpose, so that the commercially available equipment requires creative setup adaptations [24, 25]. To overcome the above limitations, we present a simplified GC–MS–EAD system, adapted from an instrument typically used in food sensory analysis, and use it to detect the VOCs emitted by a healthy dog and that elicit antennal responses in *P. perniciosus* sand flies.

## Methods

### Insect source and rearing

A collection of 1-week-old *P. perniciosus* adult females reared at the Department of Veterinary Medicine of the University of Bari [26] were used for electrophysiological tests. Newly emerged females were kept under controlled temperature (25 °C) and relative humidity (65–70%) and provided with sugar meal ad libitum until use. Experiments were always performed in the morning from 8 a.m. to 10 a.m., when sand flies were most active.

### Headspace solid-phase microextraction (HS-SPME) of dog volatile organic compounds (VOCs)

A sampling device for headspace solid-phase microextraction (HS-SPME) of dog VOCs was set up. The device consisted of a cylindrical glass chamber (7 cm height × 3.5 cm internal diameter; total volume 67.4 mL) sealed at the top by a 3D-printed polylactic acid (PLA) lid. A rubber septum (Injector Septa, Supelco, Bellefonte, PA, USA) was tightly fitted in an 8 mm hole at the centre of the lid, allowing the insertion of an SPME fibre. To prevent contamination, the inner surface of the lid was Teflon-coated. The basal edge of the glass chamber was coated with a sponge layer (0.5 cm thick) to ensure air exchange during sampling and avoid excessive pressure and discomfort to the dog.

The extraction chamber was placed on the dorsal area of the dog, and a 50/30 µm divinylbenzene–carboxen–polydimethylsiloxane (DVB–CAR–PDMS) SPME fibre (Supelco), previously conditioned for 30 min at 270 °C in the injection port of a 7890B GC (Agilent Technologies Inc., Santa Clara, CA, USA), was exposed through the septum to the headspace of the fur portion. Sampling was carried out for 30 min at 25 ± 1 °C and 65 ± 5% R.H. At the end of the exposure, the SPME fibre was retracted into its needle and kept in a thermally insulated container at approximately 10 °C before being analysed [27]. Five replicates of the SPME extracts from the same dorsal area of the dog were prepared. To confirm the absence of environmental or device-related contamination, a negative control sample was also collected by exposing the SPME fibre to the same environmental conditions and setup but in the absence of the dog.

### Gas chromatography coupled with mass spectrometry and electroantennographic detection (GC–MS-EAD)

**Fig. 1:**
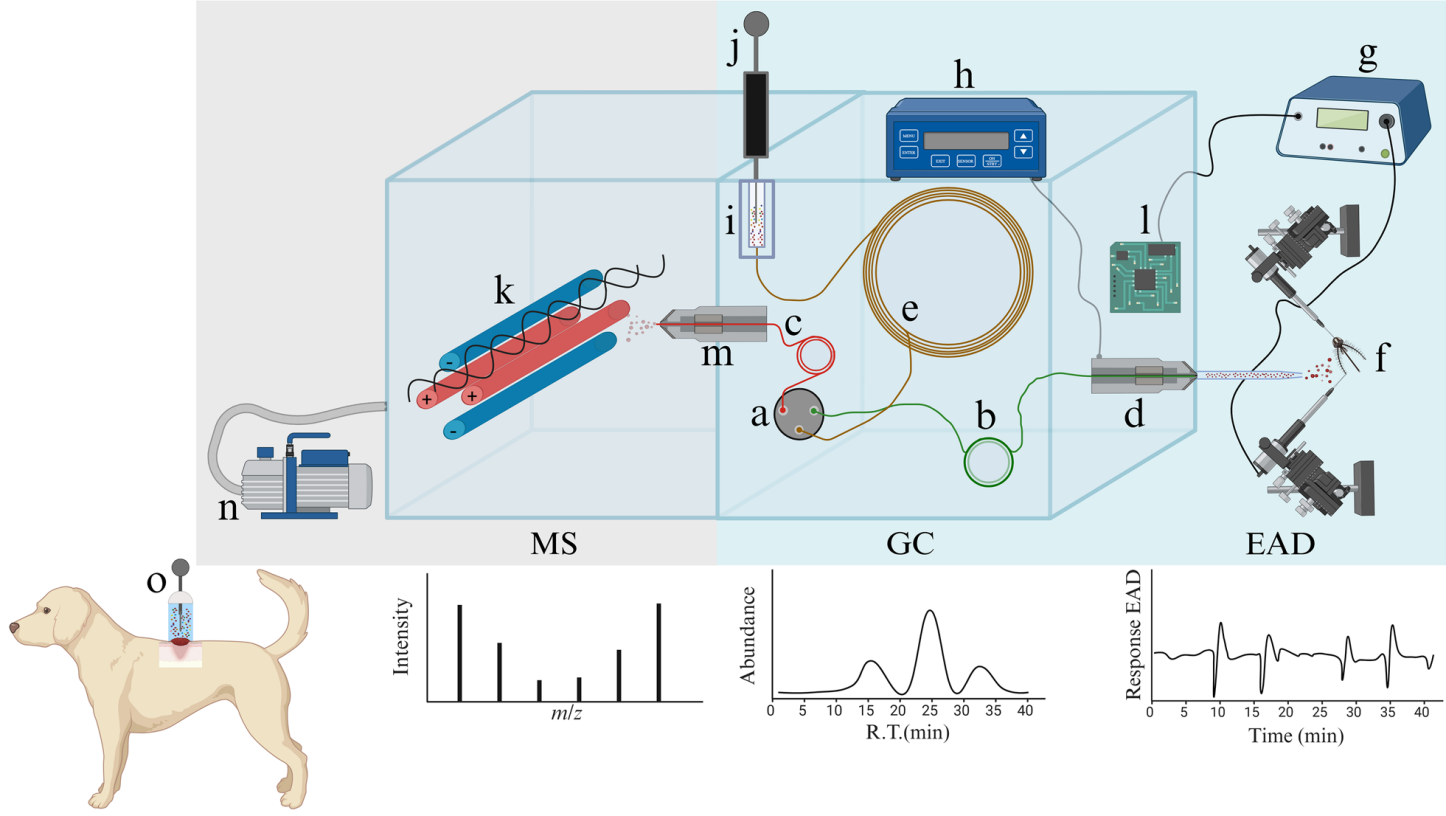
Schematic representation of the gas chromatography coupled with mass spectrometry and electroantennographic detection (GC–MS–EAD) apparatus used to detect EAD-active compounds in the headspace solid-phase microextraction (HS-SPME) extracts of volatile organic compounds (VOCs) emitted by a healthy dog. Components are labelled as follows: **a** Graphpack 3D/2 flow splitter, **b** Deactivated capillary column to the EAD, **c** Deactivated capillary column to the MS, **d** Transfer line to the EAD, **e** Main GC capillary column, **f** Antennal preparation with micromanipulators, **g** ProbeAmp signal amplifier, **h** Digital temperature controller, **i** Split/splitless injector, **j** Solid phase microextraction (SPME) fibre, **k** Quadrupole mass detector, **l** Data acquisition interface, **m** Heated transfer line to MS, **n** Vacuum pump, **o** Dog with SPME sampling device. Created in https://BioRender.com

The simplified GC–MS–EAD system set-up presented here is schematised in Fig. 1. The SPME extracts were analysed by GC–MS–EAD using the 7890B GC equipped with or without a split injector and an HP-5MS capillary column (30 m × 0.25 mm i.d. × 0.5 μm film thickness; J&W Scientific Inc., Folsom, CA, USA) and linked to a 5977 A quadrupole mass detector (Agilent Technologies Inc.). The GC conditions were previously reported [21]. Briefly, carrier gas helium at 1.25 mL/min; injector temperature at 250 °C; split time 4 min; oven program from 60 to 250 °C at 5 °C/min and then 250 °C for 15 min. The effluent from the column was equally split by a Graphpack 3D/2 flow-splitter (Gerstel, Mülheim, Germany) between the MS and a 30 cm long transfer line (Effluent Conditioner Assembly, Type EC-03, Syntech Laboratories, Hilversum, The Netherlands) by a 54.7 cm × 0.15 mm i.d. and an 83.9 cm × 0.1 mm i.d. deactivated capillary, respectively. The length and internal diameter of deactivated capillaries were determined by using the Gerstel ODP column calculator software (Gerstel). The temperature of the transfer line was regulated by a digital temperature control (Type TC-02, Syntech Laboratories) set at 250 °C. At the end of the transfer line, the column effluent was mixed to a constant flow of charcoal-filtered humidified air (500 mL/min) passing in a glass tube (6 mm i.d. × 10 cm) with the outlet positioned about 1 cm from a female *P. perniciosus* antennal preparation. In detail, the head of a 1-week-old female was excised from the thorax and mounted between two glass electrodes filled with Kaissling’s saline [28]. The indifferent electrode was inserted into the head of the insect, whereas the recording electrode was put in contact with the tip of an antenna after removing the two distal segments.

The analogue EAD signal was amplified (10 ×) and converted into a digital signal using the converter ProbeAmp (Syntech Laboratories) and recorded at 50 Hz/0.004 min rate with a data channel of the GC–MS using the GC ChemStation software in addition to the MS ChemStation software (Agilent Technologies Inc.). Five antennae of different females were used to analyse the five HS-SPME extracts. Mass spectra were recorded in the electron impact mode (i.e., ionisation energy, 70 eV) in a range of 15–550 amu at 2.9 scans/s. EAD-active compounds were identified by observing characteristic ions and by comparison of mass spectra with those of the data system library (NIST11, *P* > 90%). Moreover, the identity of EAD-active compounds was confirmed by comparing retention times and mass spectra with those of commercially available standards purchased from Sigma-Aldrich Inc. (Milan, Italy, chemical purity 80–99%). A mixture of a continuous series of straight-chain hydrocarbons, C5-C40 (Alkane Standard Solution C5-C40, Sigma Aldrich, Milan, Italy), was injected into an HP-5MS column under the same conditions previously described to obtain the linear retention indices (RIs) [29].

## Results and discussion

**Fig. 2:**
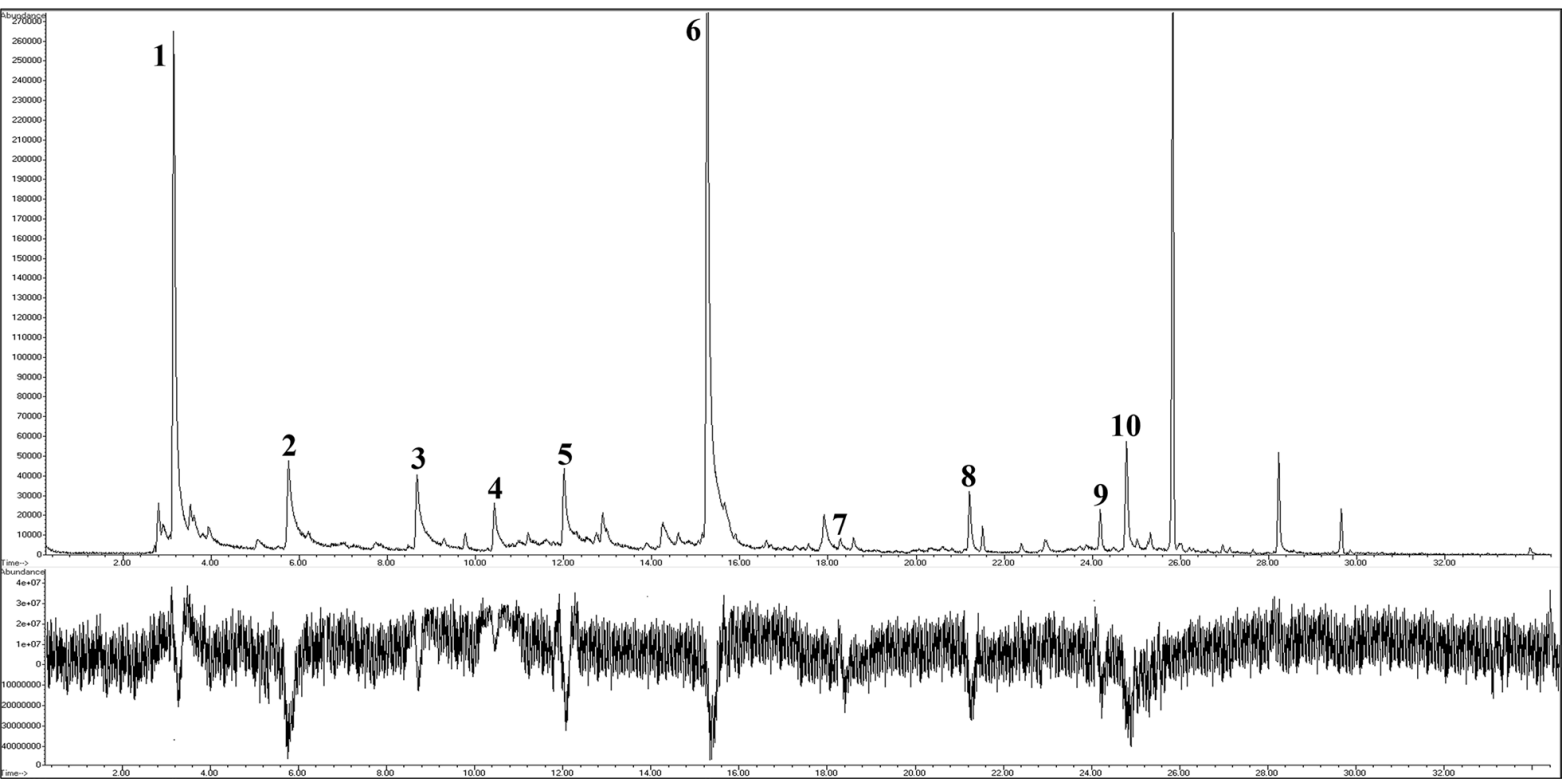
Gas chromatography–mass spectrometry–electroantennographic detection (GC–MS–EAD) analysis of *Phlebotomus perniciosus* response to volatile organic compounds (VOCs) emitted by a healthy dog

The GC–MS–EAD analysis of the HS-SPME extracts from a healthy dog revealed ten EAD-active peaks (Fig. 2), corresponding to the experimental retention indices reported in Table 1.

**Table 1:** EAD-active volatile organic compounds (VOCs) emitted by a healthy dog

Peak	^a^RI_exp_	RI_lit_	Compound	Functional group	^b^Id
1	600	600 [30]	Hexane	Alkane	RI, MS
2	800	800 [31]	Hexanal	Aldehyde	RI, MS
3	901	901 [32]	Heptanal	Aldehyde	RI, MS
4	960	962 [33]	6-methylheptan-2-one	Ketone	RI, MS
5	1003	1004 [30]	Octanal	Aldehyde	RI, MS
6	1107	1103 [32]	Nonanal	Aldehyde	RI, MS
7	1207	1203 [32]	Decanal	Aldehyde	RI, MS
8	1310	1313 [30]	Octanal, 7-hydroxy-3,7-dimethyl-	Aldehyde	RI, MS
9	1432	1428 [32]	Caryophyllene	Sesquiterpene	RI, MS
10	1455	–	2-Methylundecane-2-thiol	Thiol	MS

^a^Retention index (RI) experimental (exp) determined using an Agilent HP-5MS GC capillary column, subsequently compared with literature (lit) RI values obtained from the same or a similar type of column

^b^Compound identification criteria: RI: retention index matching, with the difference between measured and lit RI values ≤ 10 units. MS: mass spectrum matching against a reference database spectrum and authentic standards. 2-methylundecane-2-thiol was tentatively identified. References are reported in square brackets

By RI and MS matching [30–33], the peaks were identified as follows: 1. hexane; 2. hexanal; 3. heptanal; 4. 6-methylheptan-2-one; 5. octanal; 6. nonanal; 7. decanal; 8. octanal, 7-hydroxy-3,7-dimethyl-; 9. caryophyllene; 10. 2-methylundecane-2-thiol (tentative identification). The strongest antennal responses (i.e., more pronounced depolarisation) were elicited by peaks 2 and 6, namely the aldehydes hexanal and nonanal.

Numerous aldehydes, alcohols, ketones and carboxylic acids have been detected previously among the VOCs emitted by dog coats and bodies. Hair samples from healthy and *L. infantum*-infected dogs, extracted through HS-SPME and analysed by GC–MS, have shown the presence of the aldehydes octanal, nonanal, decanal [19, 20], hexanal and heptanal [19] as observed in our study. Furthermore, Holderman et al. [34] identified 182 compounds from four healthy dogs sampled by Tenax TD sorbent tubes placed on their dorsal midline, with hexanal, heptanal and octanal being the same aldehydes also detected herein. Quantitative and qualitative discrepancies can be attributed to the different methods used for VOC collection, the breed and sex of the dogs and especially, their health status. Similarly, hexanal, heptanal, octanal, nonanal and decanal have also been detected among human skin volatiles [35].

In a previous study, 28 compounds isolated from canine and human emanations were demonstrated to be perceived by (through EAG) and affect (through olfactometry) *P. perniciosus* [22]. Of the 14 semiochemicals (6 of which were aldehydes) that elicited higher antennal EAG-response, nonanal was significantly attractive in a Y-tube assay. Likewise, it proved to be one of the most EAD-active compounds in the present study.

Analogously to *P. perniciosus*, other sand flies showed the ability to perceive the same aldehydes released by vertebrate hosts. For instance, *Lutzomyia longipalpis* (Lutz and Neiva) males were activated and attracted by pure octanal, nonanal and decanal, and by a blend of octanal, decanal and heptadecane (1:1:1) in a wind tunnel assay [36]. Octanal and decanal also activated females but were not attractive [36]. Moreover, *L. longipalpis* males and females were activated and attracted in a concentration-dependent way by the alcohols 1-octen-3-ol and 1-nonanol [37]. In addition, the semiochemicals 1-hexanol, 1-octen3-ol and 1-octanol also activated the host-seeking behaviour and attracted females of the species *Nyssomyia neivai* (Pinto) [38, 39]. It should be noted that these alcohols are typical plant volatiles from fruits. Therefore, as sand flies are also glycophagous, their feeding habits may explain the attractive effect of such compounds.

To identify potential semiochemicals from an extract of VOCs, one available technique is the GC-EAD [40]. In this approach, the extract to be analysed is injected into a GC apparatus, and the different compounds contained are separated on the basis of their molecular weight, functional group and chemical affinity with the stationary phase (e.g., polydimethylsiloxane, polyethylene glycol, methyl silicone) of the GC column chosen. At the end of the elution, each compound can be divided through a flow-splitter in a 1:1 ratio, so that half of it is directed to the flame ionisation detector (FID) of the GC and half on the insect antennal preparation mounted in an EAD device. The separated compounds move along the GC column pushed by the carrier gas under atmospheric pressure in the segments joining the flow-splitter with the FID (splitter-FID on one side) or the antennal preparation (splitter-EAD on the other side), in an approximately 1:1 ratio at the same time. For each compound, the GC-EAD provides a GC profile showing the retention time and its potential electrophysiological activity. However, the identity of the compounds remain unknown, as their retention times change according to the analytical conditions set. Therefore, the extract must be further analysed through a GC–MS [40] equipped with a comparable GC column to obtain the same peak profile and sequence. With this additional analysis, we can acquire the mass spectrum of each peak to understand if it belongs to a singular or co-eluting compound and (tentatively) identify it by mass spectra comparison with known standards analysed under the same conditions.

When attempting to merge GC-EAD with GC–MS in a GC–MS–EAD system, new technical limitations arise. After the separation operated by the flow-splitter, half of the compound moves with the carrier gas under atmospheric pressure towards the antenna, but while the mass spectrometer is connected, the other half is also pushed by the vacuum generated by the MS itself. To let the compounds properly reach the two destinations (EAD and MS) at the same time and give aligned peaks (i.e., antennal response and mass spectrum), the transfer speed must be the same. Several studies have tried to solve this problem by using columns of different lengths and diameters or regulators. Initially, Weissbecker et al. [24] opted for restriction capillaries (i.e., 0.1 mm i.d. leading to MS and 0.15 mm i.d. leading to EAD) to work with the house longhorn beetle’s antennae. Starting from this original setup, other authors made minor adjustments by selecting different GC columns more suitable for their specific entomological investigations [41–48].

Instead, Schott et al. [25] used mass-flow controllers and an analogue-to-digital converter for their portable GC–MS–EAD setup to detect low ambient pheromone concentrations in vineyards. Concerning the GC–MS–EAD technique herein employed, we can consider it as a simplified version of the devices previously created. We exploited an instrument typically used to couple GC, olfactometry and MS (GC-O-MS) to characterise food aroma and flavour [49]. Basically, we replaced the ‘olfactory detector port’ (ODP-2, Gerstel) with a Syntech Effluent Conditioner Assembly (Type EC-03) regulated by a digital temperature control (Type TC-02) and mounted the electroantennographic preparation of the insect antenna in the place of the sniffing port, where the nose of a trained sensory analyst would usually act as a detector. In this system, the flow-splitter itself (Graphpack 3D/2 flow-splitter, Gerstel) balances the speed of the two outgoing streams. Furthermore, it involves the use of columns with different lengths and diameters in the two sections, splitter-MS and splitter-EAD (i.e., 83.9 cm × 0.1 mm i.d. vs. 54.7 cm × 0.15 mm i.d., respectively). Finally, unlike the previous setup in which the antenna was mounted in a polytetrafluoroethylene detector cell [24], in our system, the column effluent at the end of the transfer line is mixed with a constant air stream and directly passes over the antenna through a glass tube. These expedients, together with the antennal signal amplification through an additional device (ProbeAmp, Syntech Laboratories), allow us to record the double trace (Fig. 2) with a dedicated GC–MS software (ChemStation software, Agilent Technologies Inc.).

## Conclusions

The GC–MS–EAD technique, adapted from a food sensory analysis instrument, proved effective for detecting semiochemicals that elicit antennal stimulus in *P. perniciosus* females. Integrating chemical and electrophysiological analytical techniques reduced the costs, eliminating the need for FID, the experimental time and sample quantity needed, also minimising the risk of EAD-active peak misidentification. Its application could be further validated by other host–vector pairs, potentially advancing our understanding of their interactions and enabling strategic interventions. Future research should explore the repellence or attractiveness of the EAD-active compounds identified in this investigation and which have not been previously assessed in *P. perniciosus*. Bioassays such as Y-tube olfactometry or wind tunnel experiments using compounds such as hexane, heptanal, 6-methylheptan-2-one, octanal, 7-hydroxy-3,7-dimethyl-, caryophyllene and 2-methylundecane-2-thiol (tentatively identified) could reveal novel tools for integrated vector monitoring and control.

## Data Availability

Data supporting the main conclusions of the study are included in the manuscript.

## Abbreviations

DVB–CAR–PDMS: Divinylbenzene–carboxen–polydimethylsiloxane
EAD: Electroantennographic detector
EAG: Electroantennography
FID: Flame ionisation detector
GC: Gas chromatography
GC–MS–EAD: Gas chromatography–mass spectrometry–electroantennographic detection
HS-SPME: Headspace solid-phase microextraction
MS: Mass spectrometry
MS: Mass spectrum
PLA: Polylactic acid
RI: Retention index
VL: Visceral leishmaniasis
VOCs: Volatile organic compounds
